# Impact of Graphene Oxide on Properties and Structure of Thin-Film Composite Forward Osmosis Membranes

**DOI:** 10.3390/polym14183874

**Published:** 2022-09-16

**Authors:** Chenglong Dai, Dan Zhao, Yongqiang Wang, Rui Zhao, Han Wang, Xiangci Wu, Shejiang Liu, Huizhen Zhu, Jianfeng Fu, Mengling Zhang, Hui Ding

**Affiliations:** 1 School of Environmental Science and Engineering, Tianjin University, Tianjin 300072, China; 2 Huadian Aqua Membrane Separation Technology (Tianjin) Company Limited, Tianjin 301700, China

**Keywords:** forward osmosis, graphene oxide modification, desalination, membranes, water channels

## Abstract

Forward osmosis (FO) membranes have the advantages of low energy consumption, high water recovery rate, and low membrane pollution trend, and they have been widely studied in many fields. However, the internal concentration polarization (ICP) caused by the accumulation of solutes in the porous support layer will reduce permeation efficiency, which is currently unavoidable. In this paper, we doped Graphene oxide (GO) nanoparticles (50~150 nm) to a polyamide (PA) active layer and/or polysulfone (PSF) support layer, investigating the influence of GO on the morphology and properties of thin-film composite forward osmosis (TFC-FO) membranes. The results show that under the optimal doping amount, doping GO to the PA active layer and PSF support layer, respectively, is conducive to the formation of dense and uniform nano-scale water channels perpendicular to the membrane surface possessing a high salt rejection rate and low reverse solute flux without sacrificing high water flux. Moreover, the water channels formed by doping GO to the active layer possess preferable properties, which significantly improves the salt rejection and water permeability of the membrane, with a salt rejection rate higher than 99% and a water flux of 54.85 L·m^−2^·h^−1^ while the pure PSF-PA membrane water flux is 12.94 L·m^−2^·h^−1^. GO-doping modification is promising for improving the performance and structure of TFC-FO membranes.

## 1. Introduction

In the past few decades, researchers have done many works to deal with water pollution and water shortage problems [1,2,3]. Forward osmosis (FO), receiving great attention, utilizes the difference in osmotic pressure in the solutions on both membrane sides to transport water molecules with a high water recovery rate and low energy consumption [4,5,6,7]. Therefore, new membrane materials, such as aquaporin biomimetic membrane [8,9], carbon nanotube modified membrane [10,11,12], graphene-based material [13,14], and graphene oxide (GO) modified membrane [15,16,17,18], have been developed for water treatment by forward osmosis.

A good FO membrane material should have high salt rejection, good hydrophilicity, high water flux, high mechanical strength, and good stability, which can be affected by the active layer and support layer [19,20,21]. Generated by solute accumulating in the porous support layer, internal concentration polarization (ICP) results in membrane fouling and decreases the effective driving force of osmosis [22], which cannot currently be avoided [23]. Researchers have developed strategies such as reducing the curvature, increasing the porosity, and improving the hydrophilicity of the load layer to deal with the problem caused by ICP [24]. As one of the most promising two-dimensional nanomaterials [25,26], GO possesses good hydrophilicity, selectivity, and excellent mechanical properties [27], which can significantly improve the separation ability and filtration efficiency of the membrane and broaden the application range. Moreover, because of the oxygen-containing groups on GO surfaces, agglomeration of the nanoparticles can be avoided [28,29]. More significantly, the surface mobility of GO can be used to adjust the nanochannel at the interface, which will provide a wider application prospect for membrane modification.

GO-modified membranes have demonstrated good performance in FO processes [30,31]. Park et al. [24] studied that the coalescence of GO into the polysulfone (PSF) support layer improved the structural properties, and accordingly improved the membrane selectivity and water permeability. Jin et al. [23] studied the formation of a polyamide (PA)-GO membrane through GO cross-linking. The highly porous and hydrophilic support layer improved separation performance and reduced ICP. Hu and Mi [32] obtained a GO membrane with great organic rejection and water flux through a layer-by-layer deposition method. Choi et al. [33] successfully prepared a TFC membrane with a support layer of PSF-PDA/GO through phase inversion and interfacial polymerization, which increased water permeability with a low GO content. PA membranes, found to have good selectivity and permeability [28,34], have been combined with GO through the interfacial polymerization (IP) process to reduce reverse salt flux and increase water flux [35]. The combination of GO and PA has the ability to increase the rejection rate of organic pollutants and reduce membrane fouling [15,36].

According to published results of GO-modified membranes, GO plays a crucial role in the final performance of the membrane. To deal with the agglomeration problem and prevent swelling of GO nanoparticles [37,38], GO is generally doped in the support layer or the active layer. However, for GO-doped membranes, most of the reported works focused only on the single doping of support layers or active layers. In this study, GO nanoparticles, employed as fillers and prepared by improved Hummer’s method [39,40], were simultaneously doped in the active layer and support layer of the thin-film composite forward osmosis (TFC-FO) membrane. The main purpose of this study was to investigate the effects of GO nanoparticles on the formation of water channels and to compare the performance of FO membranes doped with GO in different layers. We first doped GO only in the PSF support layer or the polyamide (PA) active layer and obtained two membranes with a single layer doped. The two membranes were names as TFC-FO_PSF/GO_ and TFC-FO_PA/GO_. Then, GO was simultaneously doped in the support layer and active layer, and such a membrane was represented as TFC-FO_PSF-PA/GO_. The GO loadings of the three membranes were equal. The morphological structures of the three TFC-FO membranes were characterized to explore the effects of GO on the membrane structure and properties. Finally, FO experiments were performed in pressure retarded osmotic (PRO) mode (active layer toward draw solution or AL-DS) and FO mode (active layer toward feed solution or AL-FS) to investigate the performance of different membranes.

## 2. Experimental Methods

### 2.1. Material

GO with a particle size of 50–150 nm was used as the membrane doping material. Polysulfone (PSF, Acros Organics, Mw = 60,000), N-methylpyrrolidone (NMP, analytical grade), N,N-dimethylformamide (DMF, analytical reagent), n-hexane (analytical reagent), trimesoyl chloride (TMC, 98%), and m-phenylenediamine (MPD, 99.5%) were supplied by Tianjin Yuanli Chemical Co., Ltd. (Tianjin, China). Sodium chloride (NaCl, analytical grade) and glucose (C_6_H_12_O_6_·H_2_O, analytical grade) for membrane permeability testing were supplied by Tianjin Komio Chemical Reagent Co., Ltd. (Tianjin, China).

### 2.2. Preparation of PSF/GO Support Layer

PSF or PSF/GO membranes are employed as the base membrane or support layer of the final TFC-FO membranes. The phase inversion method was employed to prepare the PSF/GO support layer. GO nanoparticles were uniformly dispersed in DMF by ultrasound treatment for 1 h. Then, GO dispersion with different GO contents (0 wt%, 0.05 wt%, 0.15 wt%, 0.2 wt%, 0.3 wt%, weight ratio to PSF) was added to NMP solutions containing 8 wt% polysulfone. The prepared mixture was stirred in a water bath at 60 °C and stood for 24 h to defoam. With an automatic membrane coating machine, the membrane was coated on a glass plate to obtain a PSF/GO base membrane with a thickness of 100 μm.

### 2.3. Preparation of TFC-FO Membranes

#### 2.3.1. TFC-FO_PSF/GO_ Membrane Preparation

An interfacial polymerization (IP) method was employed to prepare the dense active layer of the membrane [41]. The prepared PSF/GO base membrane was first soaked in an MPD aqueous solution (2%) for 2 min. The membranes were then placed in a custom plexiglass container, and the relatively dense side was contacted with an n-hexane solution containing 0.1 wt% TMC for 1 min. The membranes were dried in air at room temperature and then in an oven at 60 °C to obtain the final TFC-FO_PSF/GO_ membrane. For TFC-FO_PSF/GO-0_, TFC-FO_PSF/GO-0.05_, TFC-FO_PSF/GO-0.15_, TFC-FO_PSF/GO-0.2_ and TFC-FO_PSF/GO-0.3_, GO nanoparticles were only added to the PSF support layer, and the loadings of GO (weight ratio to PSF) were 0 wt%, 0.05 wt%, 0.15 wt%, 0.2 wt%, and 0.3 wt%, respectively.

#### 2.3.2. Preparation of TFC-FO_PA/GO_ Membranes

For the membrane support layer, a PSF membrane without GO doping was prepared. TFC-FO_PA/GO_ membrane was prepared by the IP method. GO nanoparticles were first dispersed in a 2% MPD aqueous solution by ultrasound treatment. Then, the PSF base membrane was immersed in the prepared GO slurry with different GO contents (0.05 wt%, 0.15 wt%, 0.2 wt%, 0.3 wt%, weight ratio to PA) for 2 min. The subsequent preparation method was similar to the method used for TFC-FO_PSF/GO_ preparation. For TFC-FO_PA/GO-0.05_, TFC-FO_PA/GO-0.15_, TFC-FO_PA/GO-0.2_ and TFC-FO_PA/GO-0.3_, GO nanoparticles were only introduced to the PA active layer, and the loadings of GO (weight ratio to PA) were 0.05 wt%, 0.15 wt%, 0.2 wt%, and 0.3 wt%, respectively.

#### 2.3.3. Preparation of TFC-FO_PSF-PA/GO_ Membranes

The phase inversion method in Section 2.2 was used to prepare a basement PSF membrane with GO loading of 0.025 wt%, 0.075 wt%, 0.1 wt%, and 0.15 wt%. Then, the IP method in Section 2.3.2 was used to produce the active layer. In this process, the doping amounts of GO in the active layer were guaranteed to be 0.025 wt%, 0.075 wt%, 0.1 wt%, and 0.15 wt%, while the mass ratio of GO in the support layer and active layer was 1:1. TFC-FO_PSF-PA/GO-0.05_, TFC-FO_PSF-PA/GO-0.15_, TFC-FO_PSF-PA/GO-0.2_ and TFC-FO_PSFPA/GO-0.3_ mean that GO nanoparticles were added to both the active layer and support layer, and the total GO loadings in the two layers were 0.05 wt%, 0.15 wt%, 0.2 wt%, and 0.3 wt%, respectively.

### 2.4. Characterization of GO

Attenuated total reflection-Fourier transform infrared spectroscopy (FTIR, IRprestige-2, Tokyo, Japan) was used to analyze the oxygen-containing groups of GO, and a scanning electron microscope (SEM) was employed to observe the GO nanoparticle morphology. The changes in the 2 theta angle of GO were observed by XRD spectroscopy (Dmax/2200pc, Osaka, Japan). The Fourier trans-form infrared spectrometer was used to identify Graphene Oxide over the wave number range of 500–4000 cm^−1^. Field emission scanning electron microscopy (FE-SEM, s-4800, Japan) was used to observe the surface and cross-sectional morphology of fo films. First, the sample was completely dried. When observing the plane, cut the sample into appropriate sizes and paste it on the plane observation platform with conductive adhesive. When observing the section, the small sample chamber shall be brittlely broken with liquid nitrogen and pasted on the section observation platform with conductive adhesive. Next, gold spraying was performed with a gold spraying apparatus under the conditions of a gold spraying current of 15 mA and a gold spraying time of 80 s. Finally, it can be placed in SEM and observed at 5 kV.

### 2.5. Characterization of Membrane

Field emission scanning electron microscopy (FE-SEM, s-4800, Japan) at 15 kV was used to observe the surface morphology and microstructure of TFC-FO_PSF/GO_, TFC-FO_PA/GO_ and TFC-FO_PSF-PA/GO_ membranes. With a scan area of 5 × 5 µm, atomic force microscopy (AFM, Multimode 8.0, Schwalmstadt, Germany) was used to obtain the membrane surface roughness. The horizontal and vertical resolutions of AFM are 0.04 and 0.01 nm, respectively. First, the sample needs to be dried. Take a small piece of a flat sample, and paste it on the slide with double-sided adhesive tape. After that, the samples were tested using the tapping mode, and the surface topological junction of the fo film was obtained. The average roughness (Ra) and the root mean square roughness (Rq) of the film surface can be obtained through analysis of the supporting software. Moreover, we employed X-ray photoelectron spectroscopy (XPS, Delay-Line Detector, Britain) to analyze the membrane elemental composition, and optical tensiometer to investigate the surface hydrophilicity. The formation of the characteristic polyamide peaks was demonstrated with the aid of ATR-FTIR (IRprestige-2, Japan).

The PSF/GO membrane porosity (*ɛ*) was analyzed using Equation (1). A dry sample with a mass of *m*_2_ was soaked in water for 24 h at 25 °C. After removing the residual water, we obtained the mass (*m*_1_) of the wet sample. The results of the test were the average of five measurements. (PSF density, *ρ*_p_ = 1.24 g·cm^−3^; Density of water used for testing, *ρ_ω_* = 1.00 g·cm^−3^; Effective membrane area, *A*_m_ = 1.271 cm^2^)

(1) $$\varepsilon =\frac{\left(m_{1}-m_{2}\right)/\rho_{\omega }}{\left(m_{1}-m_{2}\right)/\rho_{\omega }+m_{2}/\rho_{Ρ}}$$

### 2.6. FO Performance Evaluation

Before evaluation, the FO membrane was immersed in deionized water at 25 ± 2 ℃ for 24 h. Using a laboratory-made forward osmosis test system, we tested the FO membrane salt rejection rate (*R*), reverse salt flux (*Js*), and water flux (*Jv*). The pure water permeation flux (*Jv*) under 2 bar of hydraulic pressure (ΔP) was measured using DI water as the feed solution. The flow length of the membrane tank was 60 cm with an effective membrane area of 1271 cm^2^, and the volume flow is 100 mL/min and the operation time is 1 h. While the temperature was fixed at 25 °C, pH = 7.5, the feed and draw fluids were circulated by two peristaltic pumps. For the *Jv* and *Js* analyses, DI water was used as the feed solution and 4.0 M NaCl as the draw solution. The extracted solution weight was continuously monitored using an electronic balance. For *R* analysis, we selected 0.5 M monohydrate of glucose (C_6_H_12_O_6_·H_2_O) as the draw solution and 1 mM NaCl as the feed solution. The calculations of *Jv*, *Js,* and *R* were conducted using Equations (2)–(4), respectively.

(2) $$Jv\left(L\cdot m^{-2}\cdot h^{-1}\right)=\frac{\Delta m}{\left(A_{m}\cdot \Delta t\cdot \rho_{w}\right)}$$

(3) $$Js(g\cdot m^{-2}\cdot h^{-1})=\frac{\Delta (C_{t}V_{t})}{A_{m}\cdot \Delta t}$$

(4) $$R=\left(1-\frac{C_{P}}{C_{f}}\right)\times 100\%$$

Δ*m* was the mass increase (g) of the draw solution, *ρ_w_* was the density (g/L) of water, Δ*t* was the run time (h), and *A_m_* was the effective membrane area (m^2^). *C_t_* and *V_t_* were the change of the feed solution concentration (mol·L^−1^) and the change of the draw solution volume (L), respectively. *C_f_* was the initial feed solution concentration (mol·L^−1^), and *C_p_* was the final draw solution concentration (mol·L^−1^).

## 3. Results

### 3.1. GO Characterization

The XRD pattern of GO (Figure S1a) showed that a strong diffraction peak appeared at the position of 2θ = 9.71°. The spacing of the crystal faces corresponding to this peak position is 0.90 nm, which is consistent with the interplanar spacing of GO from 0.7 to 1.1 nm. The interplanar spacing of the original graphite was about 0.34 nm, indicating that the GO layer was rich in functional groups. FTIR spectral characterization further proved the existence of oxygen-containing functional groups (Figure S1b). The surface morphology of the GO nanoparticles was observed by SEM spectroscopy. Figure S1d shows that the GO nanoparticles were completely peeled into a single layer with a smooth surface and wrinkled edges.

### 3.2. Surface Morphology and Microstructure of TFC-FO Membranes

**Figure 1:**
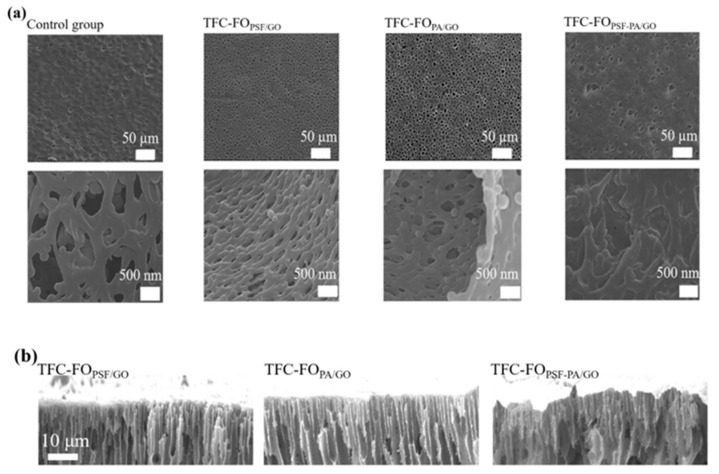
SEM images of FO membranes. (**a**) surface and (**b**) cross section.

The SEM characterization in Figure 1 demonstrates the formation of dense and uniform nano-water channels. The control group had a composite membrane without the addition of GO. The GO content of the GO doping membranes was 0.15 wt% for all characterization experiments.

As shown in Figure 1a, a porous structure was formed on the membrane surface after GO doping, reducing the ICP and facilitating water molecule transport. The diameters of the pores in the control group were 500~1000 nm; these in TFC-FO_PSF/GO_, TFC-FO_PA/GO_ and TFC-FO_PSF-PA/GO_ were 100~400 nm, 300~500 nm, and 700~2000 nm. With the enlargement of the membrane pores, we could see that the membrane pores were intertwined with numerous dense reticular channels. This unique “network structure” provided high water flux and good salt interception performance of the membranes. For TFC-FO_PSF/GO_ and TFC-FO_PA/GO_, the membrane water channels were dense and uniform after GO doping because the MPD-GO crosslinked body was crosslinked with TMC as well when the membrane contacted with TMC. However, the membrane water channels formed by doping GO in the two layers simultaneously were not fully developed, and the porosity was lower than that of TFC-FO_PSF/GO_ and TFC-FO_PA/GO_. During the IP processes for the preparation of TFC-FO_PSF-PA/GO_ membranes, the PSF-GO support layer was first reacted with the MPD-GO solution. The steric effect prevented GO nanoparticles from undergoing sufficient reactions with TMC. Therefore, more GO nanoparticles accumulated inside and at the edges of the membrane pores, which decreased the number of water channels and permeability.

In Figure 1b, the cross-sectional views indicate that all three types of GO-doped membranes have formed nano-water channels perpendicular to the membrane surface. This suggests that the surface mobility of GO with hydrophilic properties promoted the rapid exchange between nonsolvent and solvent at the phase interface. Such water channels reduced water surface resistance during the water transport process, which was beneficial for the improvement of permeability. In addition, according to Figure 1, TFC-FO_PSF/GO_ membranes had more water channels than TFC-FO_PA/GO_ membranes. The active layer of TFC-FO_PA/GO_ membrane was dense, which ensured a better salt rejection rate.

**Figure 2:**
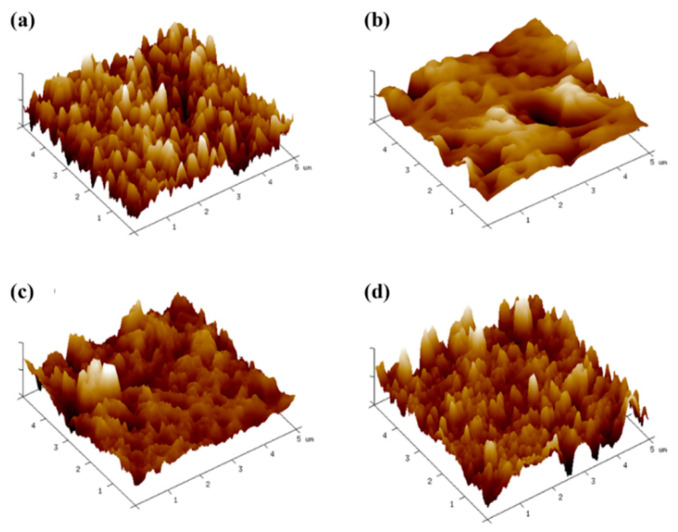
AFM diagrams of TFC-FO membranes, (**a**) GO-0, (**b**) TFC-FO_PSF/GO_, (**c**) TFC-FO_PA/GO_, (**d**) TFC-FO_PSF-PA/GO_.

The membrane surface roughness with the same doping amount was characterized by AFM (Figure 2). The TFC-FO_PSF/GO_ and TFC-FO_PA/GO_ membranes had more obvious surface roughness compared with the TFC-FO_PSF-PA/GO_ membrane as well as the membrane without GO doping (GO-0). The calculated surface roughness is listed in Table S2 with the following trend, TFC-FO_PSF/GO_ > TFC-FO_PA/GO_ > GO-0 > TFC-FO_PSF-PA/GO_. The intra-membrane mobility of GO promoted the formation of porous support layers and dense water channels in TFC-FO_PSF/GO_ and TFC-FO_PA/GO_ membranes, which increased the roughness of the two membranes. When the support layer and active layer were doped with GO at the same time, the agglomeration of GO nanoparticles might have blocked the membrane pores and was not conducive to the effective formation of the porous structure, resulting in a reduction in roughness.

Obviously, GO doping changed the surface morphology and microstructure of the TFC-FO membranes. In addition, GO contains a large number of -OH, -COOH, and other hydrophilic functional groups. After GO blending and modification, the membrane was more hydrophilic, while the interfacial reactions and polymerizations on the membrane surface were promoted accordingly.

### 3.3. Formation Mechanism of Water Channels in Membranes

We propose that the water channels in FO membranes are mainly generated in IP reaction processes. The possible reaction mechanism of the IP process used to prepare the active layers is illustrated in Figure S2. Figure S2a shows the cross-linking reaction of MPD with oxygen-containing functional groups (-COOH, -OH) of GO to form GO-MPD crosslinked bodies. In this process, amide bonds and additional hydrogen bonds are formed. As illustrated in Figure S2b,c, when the TMC/n-hexane solution was contacted with the crosslinked body, the acid chloride groups in TMC reacted not only with the amino group (-NH_2_) on MPD, but also with the oxygen-containing groups (-COOH, -OH) on the GO surface to form amide and ester groups. The GO-involved reactions prevented the agglomeration of GO nanoparticles and generated water channels in the final FO membranes.

**Figure 3:**
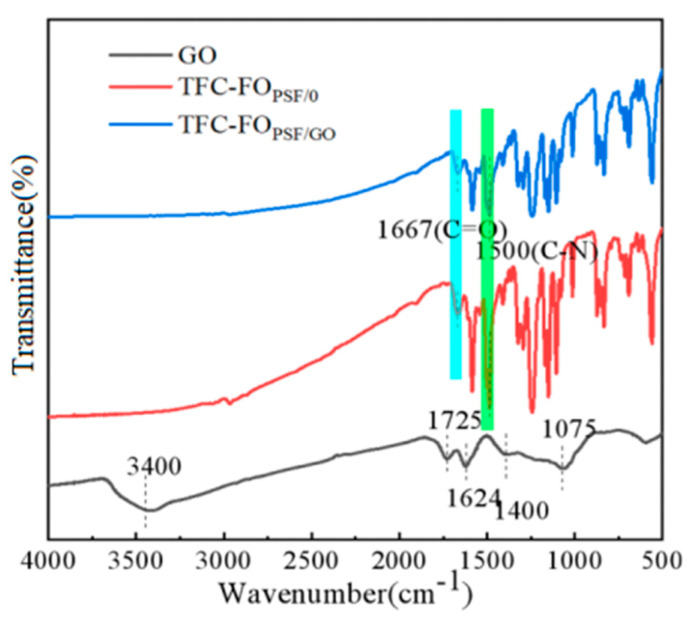
FTIR spectra of GO nanoparticles, undoped, and 0.15 wt% GO-doped TFC membranes.

FTIR spectroscopy was employed to further prove the mechanism of the IP processes (Figure 3). The obvious characteristic peaks of the amide group were observed at 1720 cm^−1^ and 1500 cm^−1^, which represented the stretching vibrations of the C = O and C-N bonds, respectively. The disappearance of -OH groups in the GO-doped TFC membrane was observed in the spectrum, which proved that GO participated in the reaction with MPD and TMC. The formation of polyamide groups promoted the formation of dense water channels. In most previous studies, the water channels in GO membranes were parallel to the membrane surface, while the GO-modified membranes studied in this paper possessed dense and uniform nano-water channels perpendicular to the membrane surface. Therefore, the proposed GO doping strategy could be used to modify the permeation performance of the membranes and reduce ICP.

**Figure 4:**
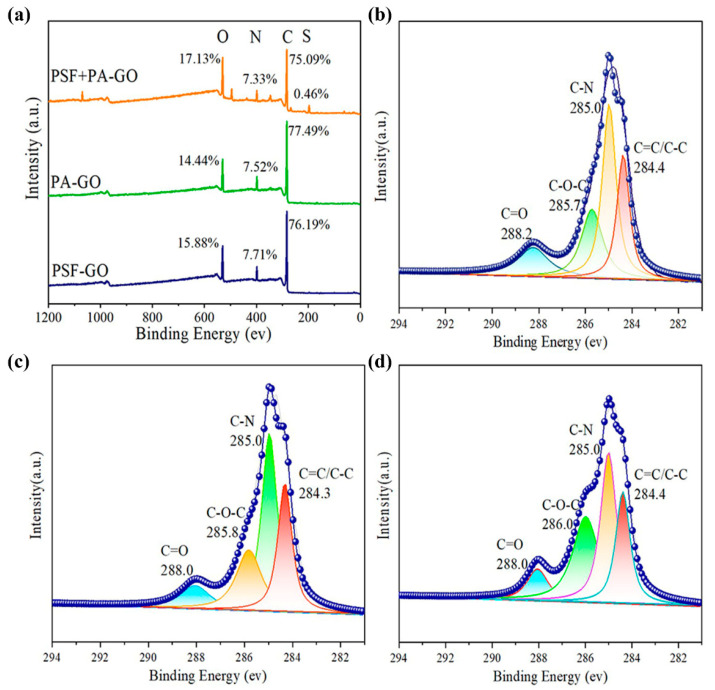
XPS spectra, (**a**) XPS full spectra, (**b**–**d**) C1s spectra of TFC-FO_PSF/GO_, TFC-FO_PA/GO_ and TFC-FO_PSF-PA/GO_ membranes.

Figure 4 illustrates the element content and chemical bond distribution of TFC-FO_PSF/GO_, TFC-FO_PA/GO_ and TFC-FO_PSF-PA/GO_ membranes. As depicted in the full XPS spectrum (Figure 4a), C, N, and O elements existed in the three types of membranes while TFC-FO_PSF-PA/GO_ contained a small amount of S element. The C1s spectrum (Figure 4b–d) mainly showed four peaks; the peaks of C = C/C-C, C-N, C-O-C and C = O were presented at around 284.4 eV, 285 eV, 286 eV, and 288 eV, respectively. According to Table S1, the percentages of C = O and C-N in the GO-doped support layer were 15.07% and 37.58%, respectively. For the GO-doped active layer, the percentages were 10.15% and 40.87%. When both layers were doped with GO, the content was reduced to 9.78% and 34.79%. Obviously, among the three cases, the GO-doped active layer had the highest ratio (4.03) of C-N to C = O. The results indicated that more nano-water channels were generated when GO was doped in the active layer, and the IP processes played a crucial role in water channel formation.

### 3.4. Effects of GO on Hydrophilicity of Different Membranes

**Figure 5:**
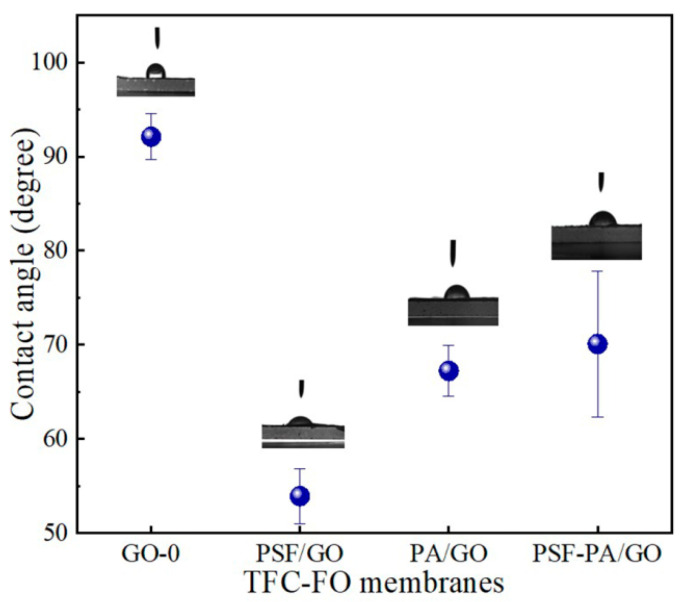
Contact angles of TFC-FO_GO-0_, TFC-FO_PSF/GO_, TFC-FO_PA/GO_ and TFC-FO_PSF-PA/GO_ membranes.

As shown in Figure 5, the contact angles of the undoped TFC-FO membrane, TFC-FO_PSF-PA/GO_, TFC-FO_PA/GO_, TFC-FO_PSF/GO_ were 92.1°, 70.07°, 67.22°, and 53.91°, respectively. Thus, GO doping had the ability to increase the hydrophilicity of the membranes and improve the permeability, while the FO membrane obtained by doping GO in the support layer showed the highest hydrophilicity. Therefore, a large number of oxygen-containing functional groups on GO surfaces enhanced membrane hydrophilicity.

### 3.5. Analyses of TFC Membrane Permeability

The effects of draw solution concentrations on water flux were analyzed first using TFC-FO_PSF/GO_ membranes and the results are presented in the Supplementary Materials. The membranes had different permeation flux under different concentrations of NaCl draw solution, and the water flux increased with the rise of the draw solution concentration because of the increased osmotic pressure.

**Figure 6:**
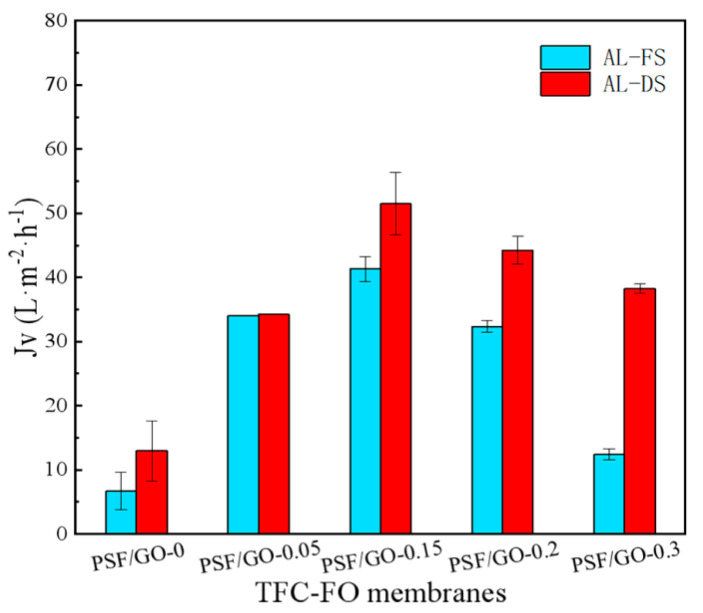
Water flux analyses in the AL-DS and AL-FS modes for TFC-FO_PSF/GO_ membranes.

For the following experiments, the feed solution was deionized water, while the draw solution was a 4 M NaCl solution. The water flux in the AL-FS and AL-DS modes was compared by testing TFC-FO_PSF/GO_ membranes with different GO loadings, and the results are illustrated in Figure 6. There was a higher water flux in the AL-DS mode experiment because the ICP was negligible when the feed solution was deionized water. In the AL-FS mode, dilution-type ICP was mainly generated. Thus, the draw solution concentration in the support layer pores decreased, which led to the decrease of *Jv* and the osmotic pressure on both membrane sides.

**Figure 7:**
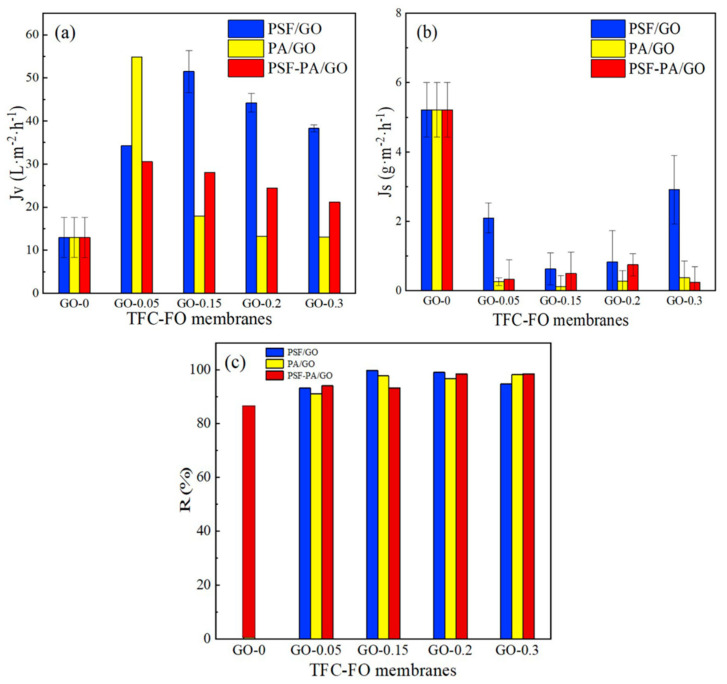
(**a**) *Jv*, (**b**) *Js*, and (**c**) *R* of different GO-doped TFC-FO membranes.

As shown in Figure 7, the *Jv, R,* and *Js* of the undoped TFC-FO, TFC-FO_PSF/GO_, TFC-FO_PA/GO_ and TFC-FO_PSF-PA/GO_ membranes were analyzed using AL-DS mode. The results indicated that modification with GO nanoparticles could significantly increase the water permeability of the TFC membrane because the formed water channels reduced water surface resistance during the water transport process. For TFC-FO_PSF/GO_ membrane, the *Jv* increased from 34.23 L·m^−2^·h^−1^ to 51.48 L·m^−2^·h^−1^ when the doping amount increased from 0.05 wt% to 0.15 wt%. Doping 0.05 wt% GO in TFC-FO_PA/GO_ led to a maximum water flux of 54.85 L·m^−2^·h^−1^. Moderate GO loading was beneficial for water channel generation. However, for membranes with high GO loadings, nanoparticles might block the water channels and pores generated in IP processes. Generally, with the increase in GO loading, *Jv* first increased and then decreased.

The *R* values of GO-doped membranes were all higher than 90% with the following trends, TFC-FO_PA/GO_ > TFC-FO_PSF/GO_ > TFC-FO_PSF-PA/GO_. For GO-doped membranes, the *R* values were all higher than 97% under the optimal doping amounts and higher than those of the control group without doping GO. Meanwhile, TFC-FO_PA/GO_ and TFC-FO_PSF-PA/GO_ membranes showed lower *Js* compared with TFC-FO_PSF/GO_. Thus, the modification of the active layer led to the smallest *Js* because the loaded GO promoted the formation of the internal structure of the membranes. GO nanoparticles were mainly bound by intermolecular forces in the GO-doped PSF support layers, and GO occupied most of the molecular gaps. When the GO nanoparticles were loaded into active PA layers, GO chemically reacted with MPD and TMC during IP processes to form polyamide groups and multi-layer GO water channels, which improved the salt interception properties of the membranes. Thus, the presence of free GO nanoparticles during the IP processes was the crucial reason for the good salt interception properties of TFC-FO_PA/GO_ and TFC-FO_PSF-PA/GO_ membranes.

When GO was doped in the two layers at the same time, *Jv* did not change significantly with the change in GO loading. *Jv* gradually decreased with the increase in GO loading because of the agglomeration of GO. In addition, GO-containing macromolecules were formed in both layers, and the IP reaction was carried out inefficiently, which hindered the formation of ordered water channels. The permeability test results were in agreement with the results of the porosity test shown in Table S3. TFC-FO_PA/GO_ membrane presented the highest porosity, while the porosity value was only 20.63% when the two layers were doped with GO simultaneously. In addition, although the porosity of TFC-FO_PSF-PA/GO_ membrane was significantly decreased after GO doping, the performance was still much better compared with the undoped membrane. Therefore, the *Jv* and salt interception performance of GO-doped membranes were mainly determined by surface morphology, polarity, and internal structure. In this study, doping an appropriate amount of GO for modifying the active layer proved to be an efficient way to establish nano water channels and improve the FO membrane performance.

**Figure 8:**
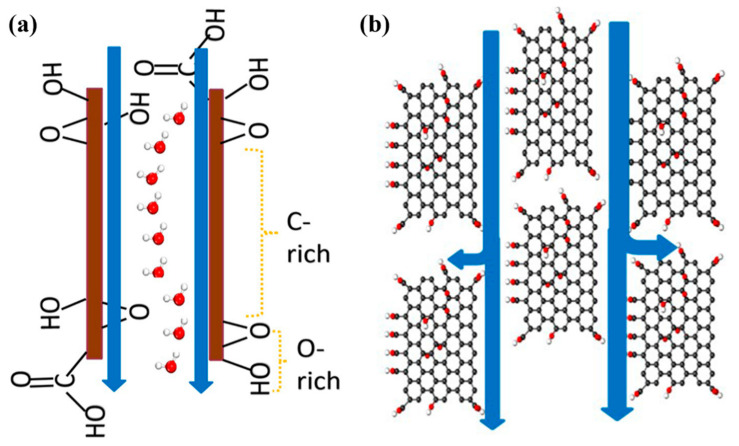
Water transport mechanism of water channels. (**a**) Single channel, (**b**) multilayer channels.

Figure 8 describes the salt–water separation mechanism of the GO-induced water channels. Figure 8a depicts the transport mechanism of a single channel. Because of the modification of GO doping, the water channels contained hydrophilic groups. Water molecules were first absorbed on the surface hydrophilic groups of the water channels. Then, the adsorbed water molecules are transmitted along the hydrophobic carbon channel perpendicular to the membrane surface for water salt separation. Figure 8b depicts the water–salt separation mechanism of the multilayer water channels that facilitated the separation of water and impurity ions, thereby improving the permeability and salt interception performance of the membrane.

## 4. Discussion

As a key component of the forward osmosis process, the structure of the FO membrane directly affects the performance and separation efficiency of the membrane [42]. To improve the performance of the FO membrane, it is very useful to modify the membrane. Doping modification is an important method for improving the structure and performance of FO membranes [43,44]. Some researchers have improved the performance of FO membranes through doping modification; for example, doping hydrophilic materials such as titanium dioxide [19], silica [45], NaY-zeolite nanoparticles [14], and functionalized carbon nanotubes [46] into FO membranes can improve the hydrophilicity and porosity of the support layer, alleviate internal concentration polarization, and increase membrane water flux. However, there were problems in that the effect was not significant, and the salt flux was generally increased.

GO shows great potential in the field of FO membrane modification due to its unique two-dimensional structure, stable chemical properties, rich oxygen-containing functional groups, and other characteristics [30,47,48]. For example, Vahid Vatanpour used polyamide (PAMAM) dendrimers to couple go to the surface of the polyamide composite (TFC) membrane, which improved the interception rate and chlorine resistance of the reverse osmosis membrane [49]; Hegab used poly L-lysine as an intermediate to graft go to the surface of the polyamide composite forward osmosis (TFC-FO) membranes, which improved the selectivity and biological pollution resistance of the membrane [36]; Hegab used polyethyleneimine and tannic acid as intermediates to coat go on the surface of TFC-FO membranes. The results showed that the live bacteria on the membrane surface were reduced by 99%, and the antibiological pollution performance of the membrane was effectively improved [30,48].

In this study, doping GO nanoparticles into the support layer or/and active layer of TFC-FO membranes was conducted to analyze the effects of GO doping on the structure and performance of the FO membranes. GO doping proved to be an efficient modification method that could significantly improve the morphological structure and increase the permeation flux of the membranes [50]. XRD and FTIR characterization proved that the GO surface contained hydrophilic oxygen-containing functional groups -OH and -COOH, which increased GO-doped membrane hydrophilicity and permeability [51], and TFC-FO_PSF/GO_ was the most hydrophilic. For the monolayer-doped GO membranes, a significantly porous structure formed on its surface, which significantly decreased ICP and promoted the transport of water molecules. However, when the support layer and active layer were doped with GO simultaneously, the membrane porous structure did not form because the steric effect made the GO nanoparticles react inadequately with TMC, and more GO nanoparticles aggregated inside and at the edges of the membrane pores, which may block the membrane pores and disfavor the effective formation of porous structures. According to the analysis of SEM images, the water channels in the composite membrane formed by doping GO into the support layer and the active layer of the FO membrane were more dense and uniform, which was better than the control group without doping GO. The water channels in the FO membrane formed by doping GO in the active layer were smaller and dense, which may be because the MPD-GO cross-linked with TMC again during the reaction with TMC, and the large gap between MPD-GO and TMC was closed during the formation of the PA active layer, a salt intercepting layer with an excellent structure formed in the membranes. However, the water channels in the composite membranes formed by doping GO into the support layer and active layer at the same time were significantly reduced, which may be because GO was first doped into the support layer and preferentially met and cross-linked with MPD-GO in the process of IP reaction. The “macromolecules” of GO nanosheets in the two reaction molecules hinder the IP reaction, resulting in more GO accumulating in the interior and edges of the membrane pores, which cannot form excellent water channels. These results are consistent with the XPS characterization results. The nano-scale water channels perpendicular to the membrane surface were formed in the prepared GO-doped membranes, reducing the resistance of water transport and accelerating rapid exchange between solvent and solute at the phase interface, which enhanced the permeability of the TFC-FO membrane. In addition, more nano-water channels are generated in TFC-FO_PA/GO._ The interfacial polymerization process is the dominant process for water channel generation [52]. In this reaction, the polyamide groups generated by the reaction of GO with MPD and TMC promoted the formation of dense water channels. The generation of water channels reduced reverse solute diffusion flux and hydraulic resistance during water transport, and also improved water flux. GO modification improved the hydrophilicity and porosity of the membranes, which improved the membrane separation performance and reduced the ICP phenomenon [53,54]. When GO particles were doped in the PSF support layer, regular, and uniform membrane channels were formed under optimal GO loading (0.15 wt%). The water channels formed by doping GO in the active layer possessed preferable properties. With the increase in GO loading, the TFC-FO membrane permeability first increased and then decreased because for membranes with high GO loadings, nanoparticles may block water channels and pores generated during IP processes [55].

## 5. Conclusions

GO doping proved to be an efficient modification method that could significantly improve the morphological structure and increase the permeation flux of the membranes. Nano-scale water channels perpendicular to the membrane surface were formed in the prepared GO-doped membranes. The interfacial polymerization process was the dominant process for water channel generation. The generation of water channels reduced reverse solute diffusion flux and hydraulic resistance during water transport, and also improved water flux. GO modification improved the hydrophilicity and porosity of the membranes, which improved the membrane separation performance and reduced the ICP phenomenon. When GO particles were doped in the PSF support layer, regular and uniform membrane channels were formed under optimal GO loading (0.15 wt%). The water channels formed by doping GO in the active layer possessed preferable properties. Doping 0.05 wt% GO in the active layer gave the FO membrane the highest *Jv* and *R,* as well as the lower *Js*, indicating the formation of dense active layers. The results indicated that GO had a decisive effect on the formation of dense active layer and porous support layer, and also improved the salt rejection rate and penetration flux of the FO membrane.

## Data Availability

The data that support the findings of this study are available from the corresponding author, Hui Ding, upon reasonable request.

## Supplementary Materials

The following are available online at https://www.mdpi.com/article/10.3390/polym14183874/s1, Figure S1: (a) XRD spectrum of GO, (b) FTIR spectrum of GO, (c) molecular structure of GO nanosheets, (d) and SEM image of GO nanosheets; Figure S2: Reaction mechanism of IP processes; Table S1: XPS results of C1s spectrum; Table S2: AFM analysis of membrane surface roughness; Figure S3: Effect of different draw solution concentrations on FO membrane water flux; Table S3: Effect of GO on porosity of TFC-FO membranes.

## References

[1] Saleem H., Trabzon L., Kilic A. (2020). Recent advances in nanofibrous membranes: Production and applications in water treatment and desalination. Desalination.

[2] Nguyen T., Lee C., Field R.W. (2020). Insight into organic fouling behavior in polyamide thin-film composite forward osmosis membrane: Critical flux and its impact on the economics of water reclamation. J. Membr. Sci..

[3] She Q., Wang R., Fane A.G. (2016). Membrane fouling in osmotically driven membrane processes: A review. J. Membr. Sci..

[4] Wang L., Li T., Chu H.Q. (2021). Natural organic matter separation by forward osmosis: Performance and mechanisms. Water Res..

[5] Wang L., Chu H.Q., Dong B.Z. (2014). Effects on the purification of tannic acid and natural dissolved organic matter by forward osmosis membrane. J. Membr. Sci..

[6] Zhao P., Gao B.Y., Yue Q.Y. (2016). Fatty acid fouling of forward osmosis membrane: Effects of pH, calcium, membrane orientation, initial permeate flux and foulant composition. J. Environ. Sci..

[7] McGinnis R.L., Elimelech M. (2007). Energy requirements of ammonia-carbon dioxide forward osmosis desalination. Desalination.

[8] Shen Y., Saboe P.O., Sines I.T. (2014). Biomimetic membranes: A review. J. Membr. Sci..

[9] Kumar M., Grzelakowski M., Zilles J. (2007). Highly permeable polymeric membranes based on the incorporation of the functional water channel protein Aquaporin, Z. Proc. Natl. Acad. Sci. USA.

[10] Liao G., Zhou X., Chen L., Zeng X., Xie X., Mai Y. (2012). Electrospun aligned PLLA/PCL/functionalised multiwalled carbon nanotube composite fibrous membranes and their bio/mechanical properties. Compos. Sci. Technol..

[11] Amini M., Jahanshahi M., Rahimpour A. (2013). Synthesis of novel thin film nanocomposite (TFN) forward osmosis membranes using functionalized multi-walled carbon nanotubes. J. Membr. Sci..

[12] Ahmed F.E., Hashaikeh R., Hilal N. (2019). Fouling control in reverse osmosis membranes through modification with conductive carbon nanostructures. Desalination.

[13] Nair R.R., Wu H.A., Jayaram P.N., Grigorieva I.V., Geim A.K. (2012). Unimpeded permeation of water through helium-leak-tight graphene-based membranes. Science.

[14] Wang L., Wu B., Liu H., Wang H., Su Y., Lei W., Hu P., Liu Y. (2019). Low temperature growth of clean single layer hexagonal boron nitride flakes and film for graphene-based field-effect transistors. Sci. China Mater..

[15] Perreault F., Tousley M.E., Elimelech M. (2014). Thin-film composite polyamide membranes functionalized with biocidal graphene oxide nanosheets. Environ. Sci. Technol. Lett..

[16] Bano S., Mahmood A., Kim S., Lee K. (2015). Graphene oxide modified polyamide nanofiltration membrane with improved flux and antifouling properties. J. Mater. Chem. A.

[17] Baig M.I., Ingole P.G., Jeon J., Hong S.U., Choi W.K., Lee H.K. (2019). Water vapor transport properties of interfacially polymerized thin film nanocomposite membranes modified with graphene oxide and GO-TiO_2_ nanofillers. Chem. Eng. J..

[18] Gao Y., Hu M., Mi B. (2014). Membrane surface modification with TiO_2_-graphene oxide for enhanced photocatalytic performance. J. Membr. Sci..

[19] Emadzadeh D., Lau W.J., Matsuura T., Rahbari-Sisakht M., Ismail A.F. (2014). A novel thin film composite forward osmosis membrane prepared from PSF-TiO_2_ nanocomposite substrate for water desalination. Chem. Eng. J..

[20] Lai G.S., Lau W.J., Gray S.R., Matsuura T., Gohari R.J., Subramanian M.N., Lai S.O., Ong C.S., Ismail A.F., Emazadah D. (2016). A practical approach to synthesize polyamide thin film nanocomposite (TFN) membranes with improved separation properties for water/wastewater treatment. J. Mater. Chem. A.

[21] Lai G.S., Lau W.J., Goh P.S., Ismail A.F., Yusof N., Tan Y.H. (2016). Graphene oxide incorporated thin film nanocomposite nanofiltration membrane for enhanced salt removal performance. Desalination.

[22] Ma N., Wei J., Qi S., Zhao Y., Gao Y., Tang C.Y. (2013). Nanocomposite substrates for controlling internal concentration polarization in forward osmosis membranes. J. Membr. Sci..

[23] Jin L., Wang Z., Zheng S., Mi B. (2018). Polyamide-crosslinked graphene oxide membrane for forward osmosis. J. Membr. Sci..

[24] Park M.J., Phuntsho S., He T., Nisola G.M., Tijing L.D., Li X., Chen G., Chung W., Shon H.K. (2015). Graphene oxide incorporated polysulfone substrate for the fabrication of flat-sheet thin-film composite forward osmosis membranes. J. Membr. Sci..

[25] Zhang K., Dwivedi V., Chi C., Wu J. (2010). Graphene oxide/ferric hydroxide composites for efficient arsenate removal from drinking water. J. Hazard. Mater..

[26] Rao C.N.R., Sood A.K., Subrahmanyam K.S., Govindaraj A. (2009). Graphene: The new two-dimensional nanomaterial. Angew. Chem. Int. Ed..

[27] Lee C., Wei X., Kysar J.W., Hone J. (2008). Measurement of the elastic properties and intrinsic strength of monolayer graphene. Science.

[28] Yang Z., Guo H., Tang C.Y. (2019). The upper bound of thin-film composite (TFC) polyamide membranes for desalination. J. Membr. Sci..

[29] Wang F., Wu Y., Huang Y. (2018). Novel application of graphene oxide to improve hydrophilicity and mechanical strength of aramid nanofiber hybrid membrane. Compos. Part A Appl. Sci. Manuf..

[30] Akther N., Phuntsho S., Chen Y., Ghaffour N., Shon H.K. (2019). Recent advances in nanomaterial-modified polyamide thin-film composite membranes for forward osmosis processes. J. Membr. Sci..

[31] Fan X., Liu Y., Quan X. (2019). A novel reduced graphene oxide/carbon nanotube hollow fiber membrane with high forward osmosis performance. Desalination.

[32] Hu M., Mi B. (2013). Enabling graphene oxide nanosheets as water separation membranes. Environ. Sci. Technol..

[33] Choi H., Shah A.A., Nam S., Park Y., Park H. (2019). Thin-film composite membranes comprising ultrathin hydrophilic polydopamine interlayer with graphene oxide for forward osmosis. Desalination.

[34] Thür R., Corvilain M., Klaysom C., Hartanto Y., Vankelecom I.F.J. (2019). Tuning the selectivity of thin film composite forward osmosis membranes: Effect of co-solvent and different interfacial polymerization synthesis routes. Sep. Purif. Technol..

[35] Shen L., Xiong S., Wang Y. (2016). Graphene oxide incorporated thin-film composite membranes for forward osmosis applications. Chem. Eng. Sci..

[36] Choi W., Choi J., Bang J., Lee J. (2013). Layer-by-layer assembly of graphene oxide nanosheets on polyamide membranes for durable reverse-osmosis applications. ACS Appl. Mater. Interfaces.

[37] Zheng S., Tu Q., Urban J.J., Li S., Mi B. (2017). Swelling of graphene oxide membranes in aqueous solution: Characterization of interlayer spacing and insight into water transport mechanisms. ACS Nano.

[38] Kim S., Ou R., Hu Y., Li X., Zhang H., Simon G.P., Wang H. (2018). Non-swelling graphene oxide-polymer nanocomposite membrane for reverse osmosis desalination. J. Membr. Sci..

[39] Dimiev A.M., Tour J.M. (2014). Mechanism of graphene oxide formation. ACS Nano.

[40] Hummers W.S., Offeman R.E. (1958). Preparation of graphitic oxide. J. Am. Chem. Soc..

[41] Choi W., Jeon S., Kwon S.J., Park H., Park Y., Nam S., Lee P.S., Lee J.S., Choi J., Hong S. (2017). Thin film composite reverse osmosis membranes prepared via layered interfacial polymerization. J. Membr. Sci..

[42] Huang L.W., Nhungoc B., Mark M., Hamlin T.J., McCutcheon J.R. (2013). Novel hydrophilic nylon 6,6 microfiltration membrane supported thin film composite membranes for engineered osmosis. J. Membr. Sci..

[43] Yu Y.B., Seo S.K., Kim I.C., Lee S. (2011). Nanoporous polyethersulfone (PES) membrane with enhances flux applied in forward osmosis process. J. Membr. Sci..

[44] Huang Y.B., Jin H.Y., Li H., Yu P., Luo Y. (2015). Synthesis and characterization of a polyamide thin film composite membrane based on a polydopamine coated support layer for forward osmosis. RSC Adv..

[45] Liu X., Ng H.Y. (2015). Fabrication of layered silica-polysulfone mixed matrix substrate membrane for enhancing performance of thin-film composite forward osmosis membrane. J. Membr. Sci..

[46] Wang Y.Q., Qu R.W., Ge Q.Q., Wang H., Xu T. (2013). Preparation of polyethersulfone/carbon nanotube substrate for high-performance forward osmosis membrane. Desalination.

[47] Hegab H.M., ElMekawy A., Barclay T.G. (2016). Single-step assembly of multifunctional poly(tannic acid)-graphene oxide coating to reduce biofouling of forward osmosis membranes. ACS Appl. Mater. Interfaces.

[48] Hegab H.M., ElMekawy A., Barclay T.G. (2015). Fine-tuning the surface of forward osmosis membranes via grafting graphene oxide: Performance patterns and biofouling propensity. ACS Appl. Mater. Interfaces.

[49] Vatanpour V., Sanadgol A. (2020). Surface modification of reverse osmosis membranes by grafting of polyamidoamine dendrimer containing graphene oxide nanosheets for desalination improvement. Desalination.

[50] Shao F.F., Su X., Shen X. (2021). Highly improved chlorine resistance of polyamide reverse membrane by grafting layers of graphene oxide. Sep. Purif. Technol..

[51] Huang X., Marsh K.L., Mcverry B.T. (2016). Low-fouling antibacterial reverse osmosis membranes via surface grafting of graphene oxide. ACS Appl. Mater. Interfaces.

[52] Morales-Torres S., Esteves C.M.P., Figueireddo J. (2016). Thin-film composite forward osmosis membranes based on polysulfone supports blended with nanostructured carbon materials. J. Membr. Sci..

[53] Zhang R., Liu Y., He M. (2016). Antifouling membranes for sustainable water purification: Strategies and mechanisms. Chem. Soc. Rev..

[54] Rana D., Matsuura T. (2010). Surface Modifications for Antifouling Membranes. Chem. Rev..

[55] Chen G., Liu R., Shon H.K. (2017). Open porous hydrophilic supported thin-film composite forward osmosis membrane via co-casting for treatment of high-salinity wastewater. Desalination.

