# Supplementary material for: Impact of Graphene Oxide on Properties and Structure of Thin-Film Composite Forward Osmosis Membranes
Source: Polymers (Basel). 2022 Sep 16;14(18):3874. doi: 10.3390/polym14183874 (PMC9506024; doi:10.3390/polym14183874)
Supplement: Supplementary file 1 [file polymers-14-03874-s001.zip › polymers-1883203-supplementary.pdf]

Impact of graphene oxide on properties and structure of thin-film composite forward osmosis membranes

Chenglong Dai [1], Dan Zhao [1], Yongqiang Wang [1], Rui Zhao [1], Han Wang [1], Xiangci Wu [1], Shejiang Liu [1], Huizhen Zhu [1], Jianfeng Fu [1], Mengling Zhang [2] and Hui Ding [1,*]

[1] School of Environmental Science and Engineering, Tianjin University, Tianjin 300072, China
[2] Huadian Aqua Membrane Separation Technology (Tianjin) Co., Ltd., Tianjin 301700, China
* Correspondence: dinghui@tju.edu.cn

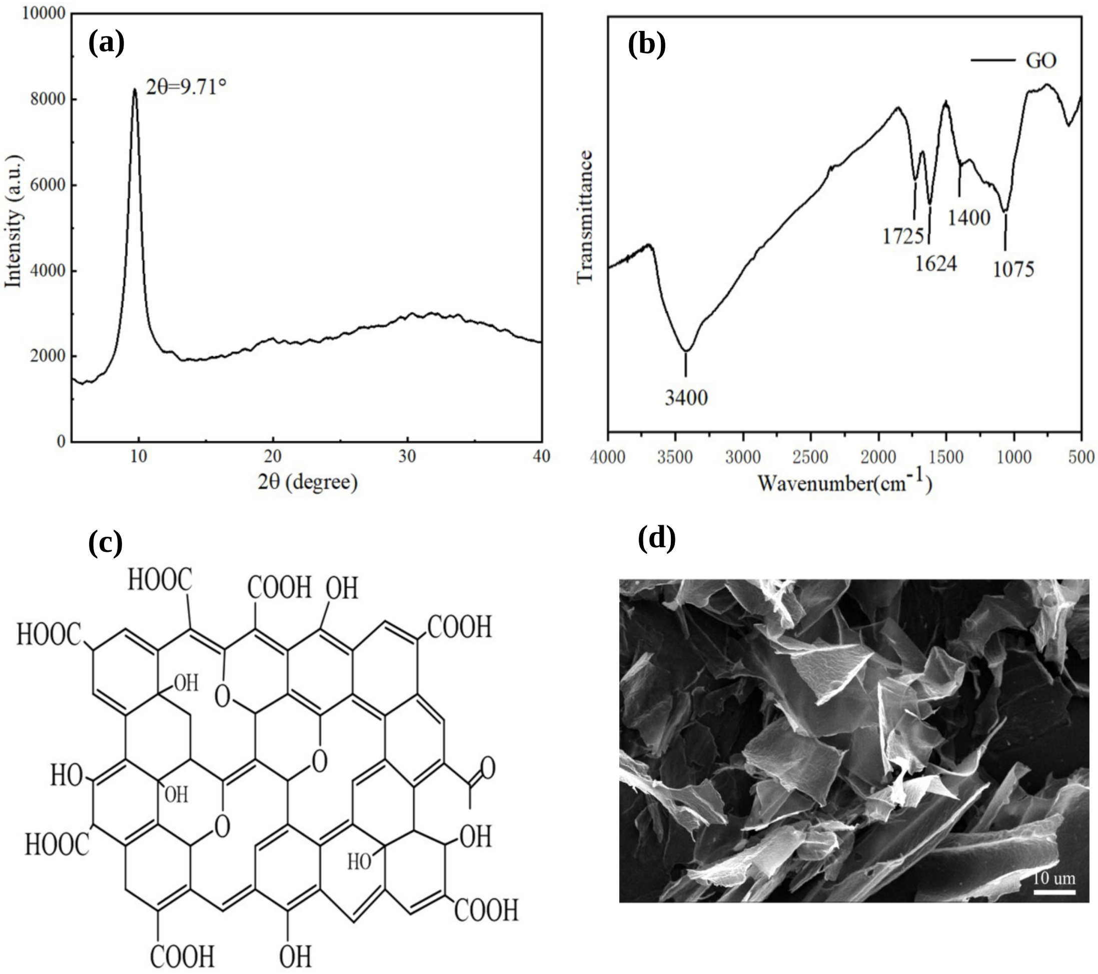

Figure S1. (a) XRD spectrum of GO, (b) FTIR spectrum of GO, (c) molecular structure of GO nanosheets, (d) and SEM image of GO nanosheets

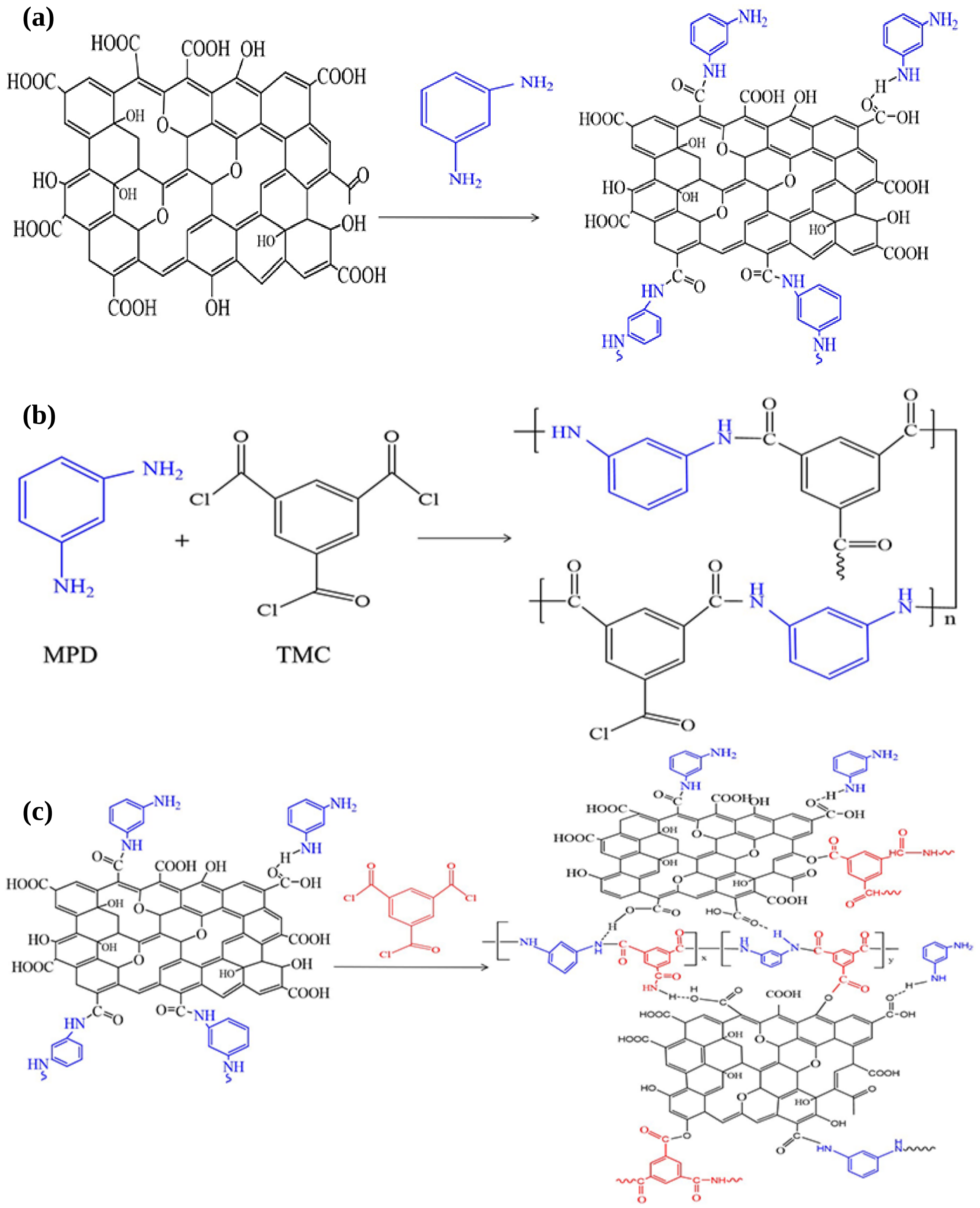

Figure S2. Reaction mechanism of IP processes

Table S1. XPS results of C1s spectrum

Membranes	C1s		
	Species	Energy (eV)	(%)
TFC-FO$_{PSF/GO}$	C=C/C-C	284.4	24.55
	C-N	285.0	37.58
	C-O-C	285.7	22.78
	C=O	288.2	15.07
TFC-FO$_{PA/GO}$	C=C/C-C	284.3	26.65
	C-N	285.0	40.87
	C-O-C	285.8	22.33
	C=O	288.0	10.15
TFC-FO$_{PSF-PA/GO}$	C=C/C-C	284.4	23.29
	C-N	285.0	34.79
	C-O-C	286.0	32.13
	C=O	288.0	9.78

Table S2. AFM analysis of membrane surface roughness

Membrane	R_a(nm)	R_q(nm)	R_{max}(nm)
GO-0	47.2	63.1	520
TFC-$FO_{PSF/GO}$	195	269	2067
TFC-$FO_{PA/GO}$	66.4	91.6	914
TFC-$FO_{PSF-PA/GO}$	34.9	47.3	551

Note*: R_a represents the average roughness of the sample measurement; R_q represents the root mean square roughness of the sample measurement; R_{max} represents the maximum roughness of the sample measurement.

Effect of draw solution concentration on water flux

The generation of osmotic pressure is closely related to the draw solution concentration. In this experiment, the feed solution was deionized water and the draw solution was 1 M, 2 M, and 4 M NaCl. 50-150 nm GO-doped FO membrane support layer was used as the test object. The membrane permeate flux was influenced by the draw solution concentration, which is shown in Figure S3. With the increase of the concentration of draw solution, the water flux gradually increases. When the concentration of draw solution reaches 4 M, the maximum water flux is obtained, which indicates that the difference of the osmotic pressure of solutions is the largest at this time.

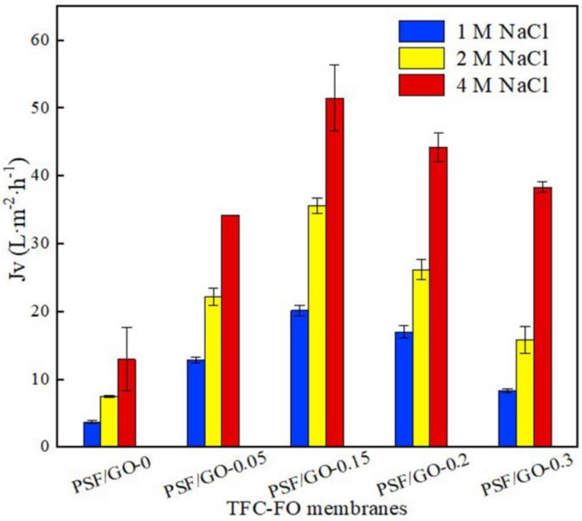

Figure S3. Effect of different draw solution concentrations on FO membrane water flux

Porosity characterization

Table S3 shows the effect of GO on the porosity of TFC-FO membranes. The porosity is highest when doping GO in the active layer, followed by doping GO in support layer, which indicated that only doping GO in the active layer or support layer can increase the porosity of the FO membranes. Compared with the control membrane, the porosity of the composite membrane doped with GO in both layers is significantly reduced, which indicates that simultaneous doping of GO in the two layers is not conducive to the formation of water channels in the membrane and may also cause membrane channel blockage. GO single-layer doping is beneficial to the formation of water channels in the membrane.

Table S3. Effect of GO on porosity of TFC-FO membranes

Membranes	Porosity/ε
$\text{TFC-FO}_{\text{GO-0}}$	62.57%
$\text{TFC-FO}_{\text{PSF/GO}}$	75.66%
$\text{TFC-FO}_{\text{PA/GO}}$	80.07%
$\text{TFC-FO}_{\text{PSF-PA/GO}}$	20.63%
